# The Accutension Stetho, an automated auscultatory device to validate automated sphygmomanometer readings in individual patients

**DOI:** 10.1038/s41371-018-0053-2

**Published:** 2018-04-06

**Authors:** Bruce S. Alpert

**Affiliations:** Department of Pediatrics, University of Tennessee Health Science Center (retired),, Memphis, TN USA

## Abstract

The aim of this report is to describe a new device that can validate, by automated auscultation, individual blood pressure (BP) readings taken by automated sphygmomanometers.The Accutension Stetho utilizes a smartphone application in conjunction with a specially designed stethoscope that interfaces directly into the smartphone via the earphone jack. The Korotkoff sounds are recorded by the application and are analyzed by the operator on the screen of the smartphone simultaneously with the images from the sphygmomanometer screen during BP estimation. Current auscultatory validation standards require at least 85 subjects and strict statistical criteria for passage. A device that passes can make no guarantee of accuracy on individual patients. The Accutension Stetho is an inexpensive smartphone/stethoscope kit combination that estimates precise BP values by auscultation to confirm the accuracy of an automated sphygmomanometer’s readings on individual patients. This should be of great value for both professional and, in certain circumstances, self-measurement BP. Patients will avoid both unnecessary treatment and errors of underestimation of BP, in which the patient requires therapy. The Stetho’s software has been validated in an independent ANSI/AAMI/ISO standard study. The Stetho has been shown to perform without difficulty in multiple deflation-based devices by many manufacturers.

Data from the Centers for Disease Control demonstrate that at least 67 million American adults have high blood pressure (BP) [1]. The diagnosis and treatment of hypertension and many other clinical conditions in which BP can be altered is dependent upon the accurate estimation of BP by indirect techniques such as oscillometry and auscultation. There are many automated sphygmomanometers available on the market, but even if the device has passed the United States National Standard protocol [2] there is no assurance that a particular device will give accurate BP readings in individual patients. To pass the auscultatory validation requirements the manufacturer must test at least 85 subjects, with a wide range of BP values needed. There are two strict statistical analyses that ensure that few readings deviate from the “gold standard” manual auscultation readings. The first criterion states that the mean ± standard deviation (SD) of at least 255 paired manual/device comparisons be less than or equal to 5 ± 8 mmHg. If one considers readings within only 2 SD values, then acceptable device values may be 16 mmHg higher, or 16 mmHg lower than the manual value. Clinicians require much more precision. It would be of great value if there is a device that could assess accuracy of automated sphygmomanometer readings on individual patients. This manuscript reports such a device. Data on validation of the app software and the generalizability of the device are also included.

The Accutension Stetho is a novel device that operates by use of a smartphone application and an inexpensive (<$30 U.S.) kit containing a specially designed stethoscope and required connectors. This report will describe the design and operation of this device.

The Accutension Stetho comes as a kit for the automated auscultatory calibration of automated sphygmomanometer (BPM) readings. The initial step is to download the “Accutension Stetho” app to a smartphone. The app contains detailed instructions/diagrams for ease of use.

Figure 1 shows the set-up for an individual patient performing the calibration of BP values from his/her self-measurement sphygmomanometer. When this procedure takes place in a medical environment the trained aide/nurse/physician would hold the smartphone. Note that the arm on which the BP is being measured is supported at heart level and the patient has back support. The patient could easily be taught to do the recording process with the trained medical personnel doing the analysis.

**Fig. 1:**
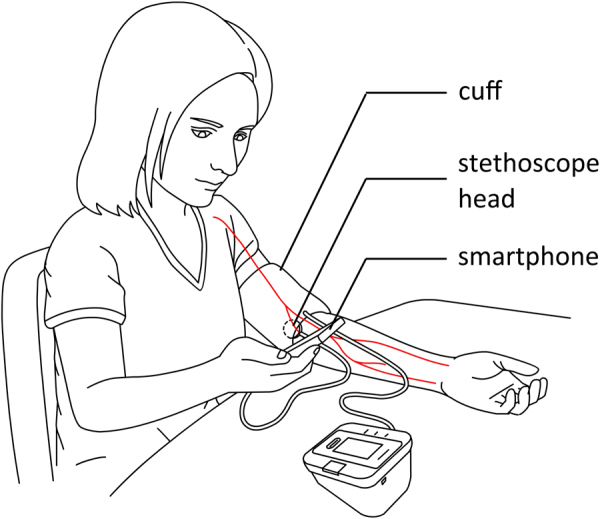
Setup and use of Accutension Stetho in a BPM self-validation

Figure 2 shows the structure of the device and demonstrates a frame of the video image on the smartphone screen during operation. The simple device hardware is made of a traditional acoustic stethoscope head (chest piece) that records Korotkoff sounds, an air tube that conducts the sounds, a microphone that converts the sounds to electrical signal, and a 3.5 mm earphone plug that transmits the electrical signal into the smartphone. During operation, the BPM is cycled, and at the end of inflation the video is begun. The patient or medical person (operator) records the BPM screen during the deflation phase. Once the deflation is complete the operator presses the stop button on the app. Note that during the BP estimation the app displays the Korotkoff sound signals and pattern. In addition, the operator may choose to listen to the Korotkoff sounds to confirm that the vertical spikes are the true Korotkoff sounds.

**Fig. 2:**
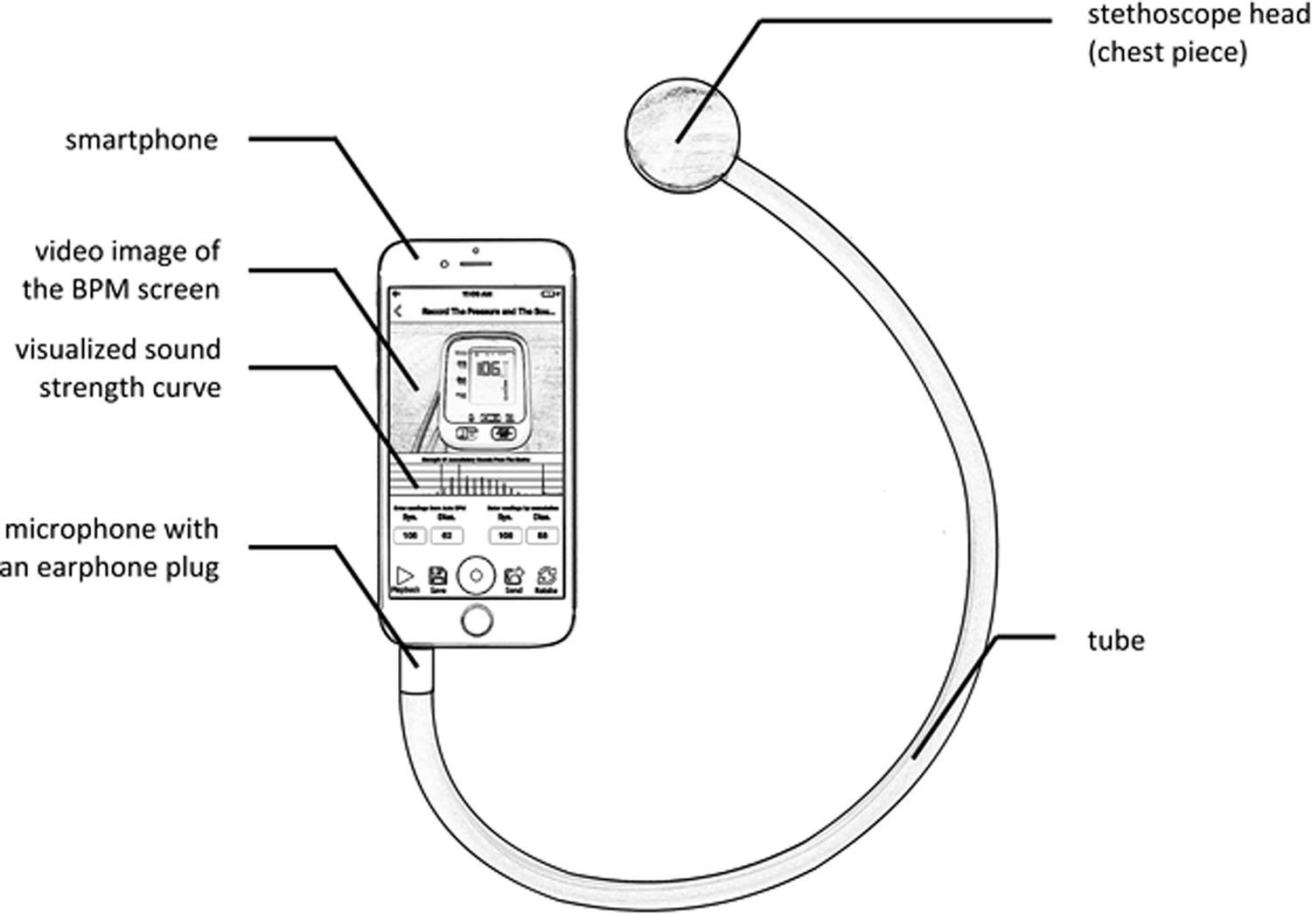
Structure of Accutension Stetho: a stethoscope consisting of a stethoscope head, a tube and a microphone with an earphone plug and an app running on the smartphone

Figure 3 displays a typical set of Korotkoff sounds [3], oscillometric pulses, and cuff pressure during a single deflation. The smartphone screen displays the Korotkoff sound profile with one vertical line that is manually movable by touch of the screen. The operator slides the vertical line to coincide with the first sharp upward spike (K1); this is the systolic BP value. Then the line is moved to coincide with the last significant sound peak (K5); this is the diastolic BP value. The app then selects the precise BP value that was displayed on the BPM screen at the time of these two selected points. If the BPM uses a continuous deflation rate the BP value on the screen will be the cuff pressure value precisely at the oscillometric spike (at the time of each Korotkoff sound deflection). If the device uses step-deflations, the value on the BPM screen will be within a few mmHg of the pressure within the cuff.

**Fig. 3:**
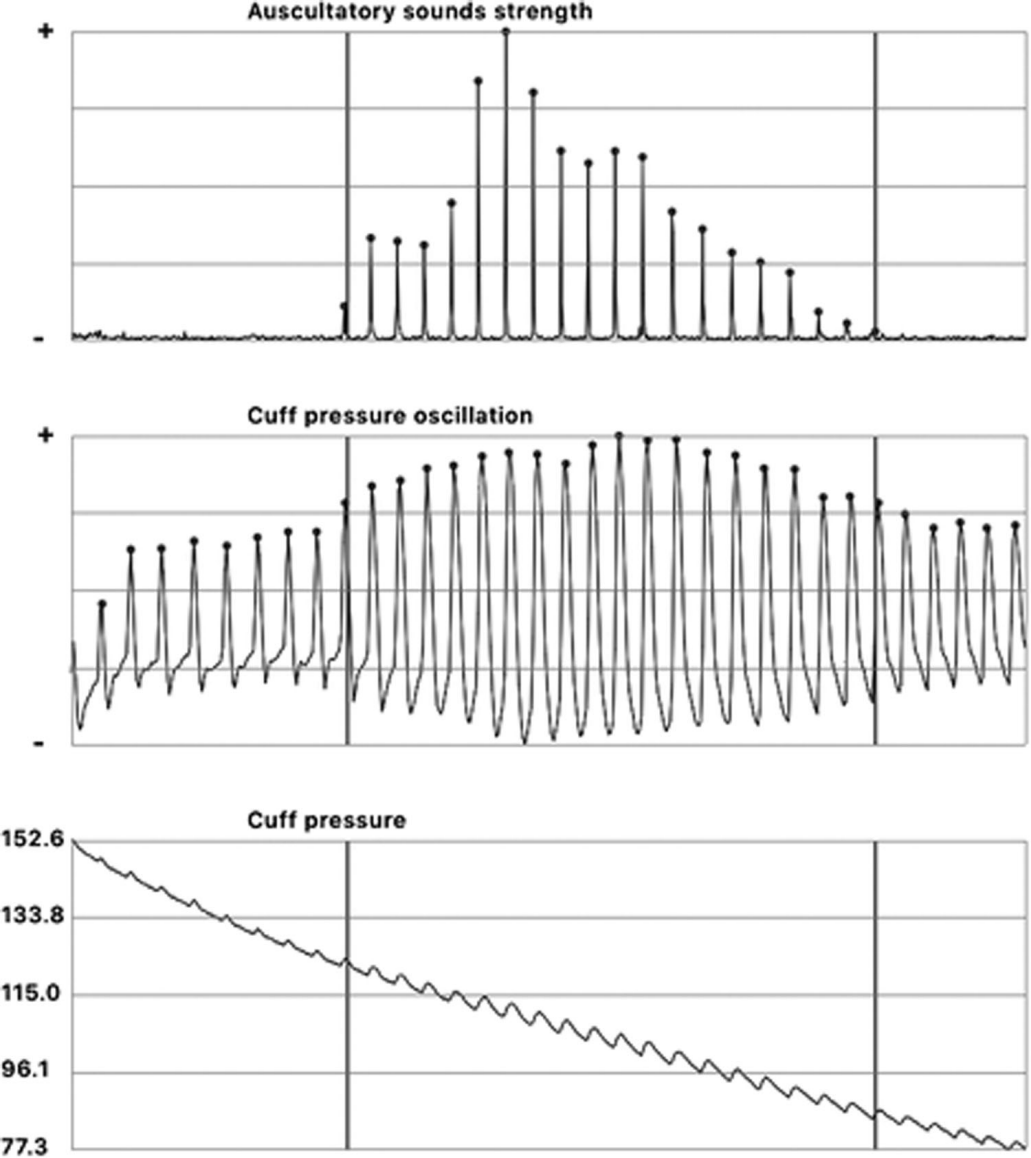
Korotkoff sounds strength, cuff pressure oscillation and cuff pressure vs. time during cuff deflation from a BPM. The dots mark the peak of Korotkoff sounds and the peak of cuff pressure oscillation, respectively, and they coincide with each other. An automated BPM refreshes the cuff pressure on its screen when it detects the cuff pressure oscillation peak

The BPM reading for an individual cycle is then compared to the reading estimated by the Accutension Stetho software. Because the Accutension detection system is by auscultation, the value will be virtually identical to the value that would have been recorded by a trained observer performing the ANSI/AAMI/ISO validation procedure [2]. A short video is attached to aid in the reader’s understanding of the operation of the process.

A recent publication by Chu et al. [4] reported the validation to the ANSI/AAMI/ISO Standard [2] requirements of an Accutension auscultatory sphygmomanometer, which is not the same device as the one described here, that uses the same software algorithm as the Accutension Stetho. The ANSI/AAMI/ISO Standard uses two methods for statistical analysis of the validation data for an automated sphygmomanometer. The mean ± SD for Method 1 (255 device minus average of two observers’ estimates) were 2.45 ± 2.24 mmHg for systolic BP and 0.69 ± 2.09 mmHg for diastolic BP. The Method 2 data (85 pooled individual patient data) showed 2.45 ± 1.47 mmHg for systolic BP and 0.69 ± 1.36 mmHg for diastolic BP. All of these values were well within the passing requirements of the Standard. The excellent correlation was in part due to the comparison of BP values recorded from manual auscultation to “simultaneous” values recorded by an automated cell phone app that also uses auscultation.

A possible limitation of the Accutension Stetho is whether it will function accurately with automated BPMs currently marketed. Table 1 displays the manufacturers and model numbers of the devices that have been tested and found to function without difficulty. The performance on deflation-based technology was consistently excellent because, even though the BPM screen displays only intermittent readings suggesting step-deflation, the software performs, in fact, linear deflation. The BP values appearing on the BPM screen are the displays of the cuff pressures at the time of an oscillometric spike, which coincides with the Korotkoff sound. The device has not been able to function when using an inflation-based technology. This issue is caused by the noise of the pump interfering with the Korotkoff sound recordings.

**Table 1:** Devices with successful testing with the Accutension Stetho

Brand	Model	Measure during deflation	Linear deflation
Omron	BP786	Yes	Yes
	BP760N	Yes	Yes
	HEM-7051	Yes	Yes
	HEM-7111	Yes	Yes
	HEM-J12	Yes	Yes
	M3	Yes	Yes
MABIS HEALTHCARE	04-596-008	Yes	Yes
HOMEDICS	BPA-060	Yes	Yes
Beurer	BM26	Yes	Yes
LifeSource	UA-767	Yes	Yes
ReliOn	BP200	Yes	Yes
Microlife	3MC1-PC	Yes	Yes
AND	UA705	Yes	Yes
Panasonic	EW3108WQ	No^a^	N/A

^a^The Panasonic device estimates BP using inflation-based technology

Automated sphygmomanometers most commonly utilize oscillometric technology. By passing the ANSI/AAMI/ISO Standard testing [2] the device can deliver accurate BP estimates to large populations. There are many variables that affect the validity of individual BP estimation, including arterial compliance, BP level, arm composition, and arm shape [3]. No manufacturer can factor into his/her algorithm corrections that would ensure BP accuracy for all patients. Thus, a device such as the Accutension Stetho will be a critical addition to the initial use of a specific BPM in a specific patient. Once a BPM has been proven to give accurate BP values the medical team can have confidence that the readings can be used for both diagnosis and treatment, if needed, for a large variety of medical conditions. Periodic revalidation should be performed to ensure that any changes in the patient’s arterial properties do not lead to BP inaccuracy. The Accutension Stetho is very inexpensive, requires minimal training, and allows medical personnel or, in some instances, the trained patient, to select a BPM for his/her care. The software algorithm utilized within the Stetho device has been validated in an independent study to meet the ANSI/AAMI/ISO Standard requirements [4]. Testing on multiple devices from multiple manufacturers has shown generalizability to deflation-based BPMs (Table 1). To date, the Stetho device has not been shown to be useful with inflation-based technology because the noise of the pump obliterates the K sounds.

The Accutension Stetho cannot, of course, correct for errors made by the medical personnel or the patient with respect to factors such as cuff size, failure to rest prior to initiating a reading, lack of arm and/or back support, etc.

The routine use of the Stetho device to ensure the accuracy of automated sphygmomanometers will improve clinical care in two ways: (1) avoiding treatment, and its possible side effects, in patients whose BP is incorrectly estimated by an automated device to be elevated, when, in fact, it is normal; (2) giving needed treatment to patients in whom the BP is incorrectly estimated by an automated device to be normal, when, in fact, it is elevated.

## Summary

### What is known about topic?

- Automated blood pressure estimation now the most commonly used methodology
- Individual blood pressure devices validated to be accurate on large populations
- Proprietary algorithms in use do not ensure accuracy for individual patients

### What this study adds?

- New smartphone app with digital stethoscope available to do automated auscultation to validate blood pressure monitor readings on individual patients
- New device critical in both clinical care for diagnosis and therapy and in research populations
- Device inexpensive and can be used by patients themselves

## Electronic supplementary material

stetho.mp4
